# Intensive care unit–acquired weakness: unanswered questions and targets for future research

**DOI:** 10.12688/f1000research.17376.1

**Published:** 2019-04-17

**Authors:** Simone Piva, Nazzareno Fagoni, Nicola Latronico

**Affiliations:** 1 Department of Medical and Surgical Specialties, Radiological Sciences and Public Health, University of Brescia, Brescia, Italy, 25123, Italy; 2 Department of Anesthesia, Intensive Care and Emergency, ASST Spedali Civili University Hospital, Brescia, Italy, 25123, Italy; 3 Department of Molecular and Translational Medicine, University of Brescia, Brescia, Italy, 25123, Italy

**Keywords:** Muscle weakness, ICU-acquired weakness, Critical Illness Polyneuropathy, Critical Illness Myopathy, muscle atrophy CRIMYNE, Critical Illness Polyneuromyopathy

## Abstract

Intensive care unit–acquired weakness (ICU-AW) is the most common neuromuscular impairment in critically ill patients. We discuss critical aspects of ICU-AW that have not been completely defined or that are still under discussion. Critical illness polyneuropathy, myopathy, and muscle atrophy contribute in various proportions to ICU-AW. Diagnosis of ICU-AW is clinical and is based on Medical Research Council sum score and handgrip dynamometry for limb weakness and recognition of a patient’s ventilator dependency or difficult weaning from artificial ventilation for diaphragmatic weakness (DW). ICU-AW can be caused by a critical illness polyneuropathy, a critical illness myopathy, or muscle disuse atrophy, alone or in combination. Its diagnosis requires both clinical assessment of muscle strength and complete electrophysiological evaluation of peripheral nerves and muscles. The peroneal nerve test (PENT) is a quick simplified electrophysiological test with high sensitivity and good specificity that can be used instead of complete electrophysiological evaluation as a screening test in non-cooperative patients. DW, assessed by bilateral phrenic nerve magnetic stimulation or diaphragm ultrasound, can be an isolated event without concurrent limb muscle involvement. Therefore, it remains uncertain whether DW and limb weakness are different manifestations of the same syndrome or are two distinct entities. Delirium is often associated with ICU-AW but a clear correlation between these two entities requires further studies. Artificial nutrition may have an impact on ICU-AW, but no study has assessed the impact of nutrition on ICU-AW as the primary outcome. Early mobilization improves activity limitation at hospital discharge if it is started early in the ICU, but beneficial long-term effects are not established. Determinants of ICU-AW can be many and can interact with each other. Therefore, future studies assessing early mobilization should consider a holistic patient approach with consideration of all components that may lead to muscle weakness.

## Introduction

Intensive care unit–acquired weakness (ICU-AW), defined as “clinically detected weakness in critically ill patients in whom there is no plausible etiology other than critical illness” ^1^, is the most common neuromuscular impairment and it affects the clinical course and outcomes of ICU patients ^2^. ICU-AW is detected in 30 to 50% of patients and the incidence is even higher (up to 67%) in critically ill patients with sepsis ^3^. ICU-AW is associated with difficulty in weaning from the ventilator, prolonged ICU stay, and higher hospitalization charges and increases long-term morbidity and mortality ^4, 5^.

**Figure 1.**
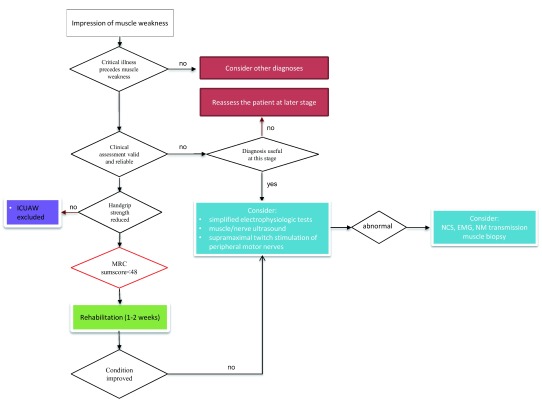
Diagnostic approach to patients developing intensive care unit–acquired weakness. EMG, electromyography; ICU-AW, intensive care unit–acquired weakness; MRC, Medical Research Council; NCS, nerve conduction study; NM, neuromuscular. Modified from Latronico and Bolton ^2^.

The term ICU-AW does not describe the condition accurately since muscle weakness is not limited to patients admitted to the ICU; indeed, it likely represents “the extreme end of a spectrum of weakness that begins with any serious illness regardless of care location” ^6^. By definition, ICU-AW is diagnosed after the onset of critical illness, which represents an important criterion to differentiate ICU-AW from Guillain–Barré syndrome or other acute neuromuscular disorders that may cause respiratory failure and ICU admission ( Figure 1 and Table 1) ^7, 8^. Weakness is symmetrical and affects all four limbs and the respiratory muscles with sparing of the facial muscles. The muscle tone is almost invariably reduced, but deep tendon reflexes can be either reduced or normal. The diaphragm is often involved, leading to prolonged mechanical ventilation and difficult weaning. ICU-AW can be ascribed to a critical illness polyneuropathy (CIP), a critical illness myopathy (CIM), or severe muscle disuse atrophy. These three conditions often coexist, and the combination of CIP and CIM – indicated as critical illness myopathy and neuropathy (CRIMYNE) or critical illness polyneuromyopathy (CIPNM) – is the most common overlap syndrome ^2^.

**Table 1.** Definition and diagnostic criteria of intensive care unit–acquired weakness, diaphragmatic weakness, critical illness polyneuropathy, critical illness myopathy, and combined critical illness polyneuropathy and myopathy.

Condition	Definition	Diagnosis
Intensive care unit–acquired weakness (ICU-AW) ^1, 2^	Clinically detected, diffuse, symmetric weakness involving all extremities and respiratory muscles arising after the onset of critical illness	c) Medical Research Council (MRC) sum score of less than 48/60 or mean MRC score of 4 in all testable muscle groups d) Dominant-hand handgrip dynamometry scores of less than 11 kg (interquartile range (IQR) 10–40) in males and less than 7 kg (IQR 0–7.3) in females
Diaphragmatic weakness (DW) ^9^	Reduced pressure-generating capacity of the diaphragm and a decreased diaphragm thickness and thickening fraction after initiation of mechanical ventilation	d) Endotracheal tube pressures less than 11 cm H _2_O after bilateral phrenic nerve magnetic stimulation during airway occlusion e) Diaphragm excursion at muscle ultrasound less than 11 mm during tidal breathing f) Diaphragm thickening fraction at muscle ultrasound less than 20%
Critical illness polyneuropathy (CIP) ^1^	An axonal, sensory-motor polyneuropathy with reduced nerve excitability and loss of axons with preserved myelin sheet	Reduced amplitude of compound muscle action potentials and sensory nerve action potentials with normal or mildly reduced nerve conduction velocity on electroneurography
Critical illness myopathy (CIM) ^1^	A primary acute myopathy with reduced muscle membrane excitability and loss of myosin filaments, fiber atrophy, and necrosis	Reduced amplitude of compound muscle action potentials and normal sensory nerve action potentials on electroneurography and reduced muscle excitability on direct muscle stimulation and myopathic motor unit potentials on needle electromyography
Combined critical illness polyneuropathy and myopathy (CRIMYNE) ^1^	Combined CIP and CIM	Reduced amplitude of compound muscle action potentials and sensory nerve action potentials combined with myopathic features on needle electromyography

CIP is a sensory-motor axonal polyneuropathy. Electrophysiological studies show a reduction in the amplitudes of compound muscle action potentials (CMAPs) and sensory nerve action potentials (SNAPs), with normal or near-normal nerve conduction velocity ( Table 1) ^2^. The histological counterpart is a primary distal axonal degeneration of motor and sensory fibers, which may cause muscle denervation and atrophy ^2^. CIM is an acute primary myopathy (that is, not related to denervation) with distinctive electrophysiological (low-amplitude motor unit potentials with early or normal full recruitment, with or without fibrillation potentials and increased CMAP duration with normal SNAPs) and morphological (loss of thick myosin filaments, muscle fiber atrophy, and necrosis) findings ( Table 1) ^2^. Muscle atrophy is the consequence of muscle unloading/inactivity, which promotes muscle catabolism that exceeds the loss in muscle cell size, resulting in decreased myocyte-specific force ^10^. Mechanical silencing – that is, the complete loss of mechanical stimuli in ICU patients who are mechanically ventilated or deeply sedated or receiving neuromuscular blocking agents or who are undergoing a combination of these – causes even more severe muscle wasting ^11^.

Although ICU-AW was defined years ago ^1^, many aspects concerning its diagnosis and its correlation with diaphragm weakness, delirium, nutritional status, and early mobilization in the ICU remain poorly understood. This review discusses these open issues that have not been completely defined and that should be addressed in future studies.

## Diagnosis of ICU-AW

ICU-AW is a clinical diagnosis ( Table 1). The Medical Research Council sum score (MRC-SS) and handgrip dynamometry constitute the gold standard for diagnosis. With MRC-SS, muscle strength is assessed in 12 muscle groups and then individual scores are combined into a sum score, which yields an overall estimation of motor function. Summed scores below 48 out of 60 and below 36 out of 60 indicate significant ^1^ and severe ^12^ weakness, respectively. With handgrip dynamometry, the isometric muscle strength of the dominant hand is measured. Cutoff scores for ICU-AW are less than 11 kg (interquartile range (IQR) 10–40) in males and less than 7 kg (IQR 0–7.3) in females ^8^. Handgrip dynamometry and MRC-SS can be used serially, and dynamometry serves as a quick screening test that, if normal, excludes ICU-AW ^13^. If abnormal, the MRC-SS is necessary to specifically identify the typical ICU-AW distribution of muscle weakness. Both tests are volitional tests that require the patient to be alert, cooperative, and motivated. As such, because of delirium, coma, pain, and the use of sedative drugs, they often cannot be used in the ICU ^14^. In these cases, non-volitional tests can provide useful clues to the diagnosis.

Simplified electrophysiological tests are non-volitional tests that have long been shown to be able to predict long-term disability in survivors of critical illness. In 1995, Leijten ^15^ first demonstrated that patients with abnormal electromyography in the ICU had persistent disability at 1 year. The simplified peroneal nerve test (PENT) has been validated in two multicenter prospective studies in Italy – the CRIMYNE-1 ^16^ and CRIMYNE-2 ^17^ studies – as a high-sensitivity test with good specificity (100% and 85%, respectively) and can be used as a screening test to identify CIP or CIM ( Figure 1). Recently ^18^, PENT was confirmed to have high sensitivity (94%, with only one false-negative result out of 72 patients examined) and excellent specificity (91%). Combined unilateral peroneal (motor) and sural (sensory) nerve assessment also has 100% sensitivity; moreover, abnormally reduced sensory and motor nerve amplitudes are associated with increased hospital mortality ^19^ and severe physical dysfunction at hospital discharge ^20^. In a large sub-study of 730 patients in the EPaNIC (Early Parenteral Nutrition Completing Enteral Nutrition in Adult Critically Ill Patients) trial, an abnormal motor nerve action potential amplitude measured at 8 days after ICU admission was independently associated with increased 1-year mortality ^21^.

Non-volitional methods with supramaximal electrical or magnetic twitch stimulation of peripheral motor nerves – that is, ulnar nerve stimulation for the adductor pollicis muscle, femoral nerve for quadriceps muscle, peroneal nerve stimulation for ankle dorsiflexor muscles, or phrenic nerve stimulation for diaphragm ^22^ – can be used to provoke muscle contraction, providing a measure of muscle function regardless of whether the patient is awake and cooperative. With transcutaneous neuromuscular electrical stimulation in healthy subjects ^23^, ramp stimulations starting from low frequencies (1–2 Hz) up to tetanic stimulation (30–50 Hz) provide a force-frequency relationship, which is a recognized method to assess the contractile properties of skeletal muscles without the need for voluntary muscle activation. Muscle ultrasound is rapidly gaining popularity among ICU physicians as a non-invasive method to assess changes in limb muscle mass as well as structural muscle alteration such as myofiber necrosis, fatty muscle infiltration, or fasciitis ^4, 24^. Abnormal echogenicity may be associated with a reduced likelihood of discharge to home, fewer ICU-free days, and increased ICU mortality ^18^. Muscle ultrasound, however, does not discriminate between patients with and those without ICU-AW at the time the patient awakens ^25, 26^. Nerve ultrasound has been shown to be a reproducible tool for diagnostics in routine clinical practice in patients with chronic inflammatory demyelinating polyneuropathy, multifocal motor neuropathy, or chronic idiopathic axonal polyneuropathy, but its use in ICU patients has not been systematically assessed ^27^.

## Diaphragmatic weakness and ICU-AW: different clinical entities or two sides of the same coin?

Diaphragmatic dysfunction or weakness (DW), defined as a decrease of diaphragm strength after initiation of mechanical ventilation ( Table 1), is common in ICU patients and, with modern technology, is easily documented ^28^. The inactivity of the diaphragm rather than mechanical ventilation per se seems to be the critical determinant of DW ^9^. Historically, concurrent limb muscle weakness or paralysis and respiratory muscle weakness causing failure to wean the patient from the ventilator have been considered pathognomonic presentations of the syndrome ^29^. However, recent studies show that DW is poorly correlated with ICU-AW ^26^ and that DW is twice as frequent as ICU-AW ^30^, favoring the hypothesis that weakness of the diaphragm and limbs might represent two distinct entities.

Several techniques are available for assessing diaphragm muscle function and these are reviewed elsewhere ^31^. When the level of endotracheal tube pressure (Pet,tw) induced by bilateral phrenic nerve magnetic stimulation during airway occlusion is used ( Table 1), DW is established if the Pet,tw falls below 11 cm H _2_O. With this criterion, DW is described in up to 64% of patients within 24 hours after intubation ^28^. DW is documented in 63 to 80% of patients at the time of weaning and in about 80% of patients requiring prolonged mechanical ventilation ^28^. When ultrasound definitions are used ( Table 1), DW is identified if the diaphragm excursion is less than 11 mm or the diaphragm thickening fraction is less than 20%. With these criteria, the prevalence of DF is lower, ranging between 29% in patients submitted to the first spontaneous breathing trial and 36% at extubation ^28, 31, 32^.

Pathophysiological mechanisms of DW are usually classified as infection/inflammation-related or ventilator-induced mechanisms ^33, 34^. Infection causes cytokine release, which in turn induces mitochondrial free radical production ^35, 36^, contributing to the reduction in muscle endurance and strength ^36^. Histopathological findings include injury of the muscle fibers with disrupted sarcomeres ^37, 38^. Controlled mechanical ventilation with complete diaphragm unloading causes marked atrophy of human diaphragm myofibers within hours ^38– 40^. Conversely, excessive diaphragm loading is associated with high levels of inspiratory effort with increased diaphragm myofiber inflammation, edema, and injury ^32^. Although disuse atrophy and muscle fiber injury are probably linked, they represent two different insults to the diaphragm and the latter seems to be an earlier phenomenon ^37^.

Pathophysiology and risk factors such as immobility and inflammation are common to both ICU-AW and DW. Histopathological features are also similar, although muscle necrosis is highly prevalent in ICU-AW ^41, 42^ but not in DW. Regardless of the pathogenesis, DW is a marker of severity of critical illness and portends a poor prognosis. If diagnosed at an early stage of acute disease, DW is associated with increased mortality ^28^. With a later onset, it is strongly associated with weaning failure ^32^, a high risk of hospital readmission in patients with chronic respiratory failure ^43^, and increased 1-year mortality ^44^.

Different strategies can be implemented to prevent DW. First, it is helpful to implement diaphragm-protective mechanical ventilation by maintaining inspiratory efforts throughout a spontaneous breathing trial ^31^ unless high respiratory drive is required. Inspiratory muscle training – via isocapnic and normocapnic hyperpnea, inspiratory resistive training, threshold pressure training, or adjustment of ventilator pressure trigger sensitivity—has been shown to have a positive impact on (1) improving inspiratory muscle strength, (2) increasing success of weaning, and (3) reducing hospital and ICU length of stay (LOS) ^45^. Muscle training applied to patients after a successful spontaneous breathing trial may increase inspiratory muscle strength and quality of life ^46^. Several drug investigations of, for example, drugs that inhibit proteolytic pathways or enhance protein synthesis ^33^ or inhibit the phosphodiesterase PDE3 and PDE4 (theophylline, 1,3-dimethyl- xanthine) ^28^ are under way. In mice, β-hydroxy-β-methylbutyrate (HMB), a leucine metabolism product that reduces muscle protein breakdown, completely prevents the dramatic decrease in diaphragm force generation caused by sepsis at dosages comparable to those used to reduce protein breakdown in human studies ^47^. In a small randomized clinical trial of 30 healthy subjects, levosimendan, a calcium sensitizer that improves cardiac contractility in patients with acute heart failure, prevented the loss of twitch diaphragm contractility after loaded breathing compared with placebo ^48^.

## Delirium, drugs, and ICU-AW

Delirium is defined as a disturbance of attention, awareness, and cognition which develops over a short period of time (hours to days) and fluctuates over time ^49^. Delirium is a severe complication in critically ill patients as it represents a decompensation of cerebral function – an “acute brain failure” – in response to one or more pathophysiological stressors.

With a prevalence rate ranging between 20 and 40%, delirium, particularly hypoactive delirium, is associated with deleterious clinical outcomes, including prolonged mechanical ventilation, increased ICU and hospital LOS, increased mortality, and impaired cognitive function for up to 12 months after discharge ^49– 51^.

Although they are clearly distinct entities, delirium and ICU-AW are possibly related and may even interact negatively with each other ^4^. Both are influenced by the severity of illness, are aggravated by the treatment adopted in the ICU, and may share some predisposing and trigger factors ( Supplementary Table 1). Disease severity at ICU admission assessed with APACHE II (Acute Physiology and Chronic Health Evaluation II) score is a predisposing factor for both conditions ^52, 53^. Benzodiazepines are strongly associated with delirium ^53^ and, by causing immobility, may increase the risk of ICU-AW ^4^. Propofol and benzodiazepines, the commonest sedative drugs used in the ICU, also directly decrease muscle excitability, worsening the effect of bed rest. Barbiturates and ketamine interact with *N*-methyl-D-aspartate receptors ^54^, which have an important role in maintaining muscle trophism ^55^.

A clear association between delirium and ICU-AW has not been established. The MOSAIC (Measuring Outcomes of Activity in Intensive Care) study (ClinicalTrials.gov Identifier: NCT03115840) is a prospective cohort study designed to assess the relationship between activity and long-term disability. When concluded (the expected date is 2020, Nathan E. Brummel, personal communication), this study will help clarify the relationship between physical activity, delirium, and cognitive dysfunction in survivors of critical illness. No study of patients with hypoactive delirium has explored the causes of reduced or absent mobility, whether it is the consequence of central nervous system or of central and peripheral nervous system and muscle dysfunction.

## Muscle metabolism, nutrition, and ICU-AW

Nutritional status is associated with weakness: starvation in healthy volunteers causes loss of muscle mass, strength, and function ^4^. Critical illness is characterized by severe skeletal muscle loss in the early stage of the ICU stay (when measured by ultrasound rectus femoris cross-sectional area) ^42^ as well as hyperglycemia and low circulating amino-acid levels ^56, 57^. The hallmark of critical illness–associated muscle wasting is the catabolic state associated with depressed anabolism ^42^. Caloric and protein supplementations do not improve the catabolic state during the early phase of critical illness, as macronutrient deficit is well tolerated compared with early caloric parenteral substitution ^58^. Protein synthesis remains refractory to increased protein delivery ^42^. Although supplementation of high doses of amino acids is safe and can be well tolerated even in patients with renal failure ^59^, results from randomized controlled trials comparing high versus low protein supplementation have yielded inconsistent results ^60– 62^.

Recent insight into the glucagon pathophysiology suggests that an elevated level of this hormone during critical illness increases hepatic amino-acid catabolism ^63^, inducing hypo-aminoacidemia. Interestingly, infusion of amino acids by raising the level of glucagon increases amino-acid breakdown in the liver, aggravating rather than reversing catabolism ^63^. Moreover, skeletal muscle wasting in critical care is directly related to impaired lipid oxidation and reduced ATP, creatine, and phosphocreatine availability induced by muscle inflammation ^64^. Mitochondrial dysfunction and ATP depletion are observed also in nerve axons and may represent a generalized phenomenon during critical illness ^65^. No study has assessed the impact of various nutritional strategies and regimens on ICU-AW as the primary outcome. In a sub-study of the EPaNIC trial ^58^, weakness assessed at an early stage of disease was significantly more common in patients receiving early parenteral nutrition compared with those receiving late parenteral nutrition but this effect was of short duration and difference was no longer significant at a later stage. Thus, the interactions between nutrition and ICU-AW remain incompletely understood ^66^.

## Early mobilization and ICU-AW

Early mobilization in the ICU has been advocated as a therapeutic strategy to prevent ICU-AW, reducing the negative effects of immobility on muscles and other organ systems ^67^. Mobilization in the ICU is feasible and safe provided that consensus guidelines are followed ^68, 69^. The incidence of potential safety events is low – cumulative incidence 2.6%, hemodynamic events 3.8, 95% confidence interval (CI) 1.3 – 11.4 and desaturation 1.9, 95% CI 0.9 – 4.3 per 1,000 mobilization/rehabilitation sessions – and medical consequences are rare (0.6% of 14,398 mobilization/rehabilitation sessions) ^70^. Evidence of efficacy, particularly long-term efficacy, remains uncertain. A recent meta-analysis of 14 randomized clinical trials enrolling 1753 patients showed no impact of active mobilization and rehabilitation in the ICU on short-term and long-term mortality, patient functional status, quality of life, ICU or hospital LOS, duration of mechanical ventilation, or discharge disposition ^71^. Patients receiving active mobilization and rehabilitation in the ICU had improved muscle strength at ICU discharge and improved walking ability without assistance at hospital discharge and more days alive and out of hospital at 6 months. In a subgroup analysis ^71^, patients receiving high-dose rehabilitation had improved quality of life in the role physical and role emotional domains compared with those receiving low-dose rehabilitation. In the recent EPICC (Extra Physiotherapy in Critical Care) trial ^66^, a 90-minute physical rehabilitation per day did improve physical outcomes at 6 months compared with 30 minutes per day, but rehabilitation started on about day 8 and the difference in terms of physical therapy actually received by the two groups was negligible (10 minutes) ^72^. Late initiation ^73^ may reduce the efficacy of mobilization as the beneficial effects of physical therapy have been found in studies in which the treatment was started early after ICU admission ^72^; however, definition of “earliness” remains undefined ^67^. In a recent randomized clinical trial, very early initiation of in-bed leg cycling and electrical quadriceps stimulation within a median of 30 hours of ICU admission did not improve global muscle strength (MRC score) at ICU discharge ^74^. The efficacy of active rehabilitation in the general ward after ICU discharge is also uncertain ^73, 75– 77^.

In stroke patients, early mobilization was demonstrated to reduce the odds of a favorable outcome at 3 months ^78^; however, the adoption of optimized session frequencies with increased daily frequency of mobilization sessions may be associated with improved outcome ^79^. Moreover, data in stroke patients may not apply to critically ill neurological patients admitted to the ICU; indeed, early mobilization is safe in this setting and might be beneficial ^80, 81^ because immobility is a common consequence of neurological impairments. A recent post-hoc analysis of a randomized controlled trial also showed that early, goal-directed mobilization is not harmful in patients with impaired consciousness and might be effective in achieving higher mobility levels and better functional status at hospital discharge ^82^.

## Conclusions and Future directions

ICU-AW is a common complication in ICU patients and has a clinically relevant impact on short- and long-term outcomes. Several important questions remain unanswered concerning the optimal method for diagnosis and the relationship between ICU-AW and DW, delirium, muscle metabolism, and nutrition. The roles of early mobilization and rehabilitation in the ICU also remain to be elucidated. Future longitudinal studies should confirm the predictive ability of early abnormalities of electrophysiological tests of peripheral nerves and muscles, muscle ultrasound imaging, and non-volitional muscle strength measurements on long-term physical dysfunction. Future efficacy nutrition trials should consider ICU-AW a clinically relevant outcome measure. Individualized timing of protein administration ^83^ should also be considered in future research studies aiming at assessing the impact of specialized nutritional strategies or regimens on ICU-AW ^84^. The overall impact of ICU mobilization and rehabilitation needs to be assessed with standardization of the optimal timing, dosage, progression of exercise, and intensity and duration of physical therapy using a core set of long-term outcome measures collected at consistent times ^4, 85^. Inclusion of mobilization and rehabilitation programs into a coordinated series of interventions such as the ABCDEF bundle with optimal pain treatment, minimal sedation, and daily spontaneous breathing trial would also be important to consider in future efficacy studies ^53, 86^.

## Supplementary material

**Possible risk factors for delirium and Intensive care unit–acquired weakness (ICU-AW).**
