# Supplementary material for: Intensive care unit–acquired weakness: unanswered questions and targets for future research
Source: F1000Res. 2019 Apr 17;8:F1000 Faculty Rev-508. [Version 1] doi: 10.12688/f1000research.17376.1 (PMC6480958; doi:10.12688/f1000research.17376.1)
Supplement: Supplementary file 1 [file f1000research-8-19002-s0000.tgz › 560fcb33-6146-479f-b471-fd37ed0f6eaa_NEW_Latronico_Supplementary_Table_1_FM_refs_fixed.docx]

**Supplementary Table 1.** Possible risk factors for delirium and Intensive care unit–acquired weakness (ICU-AW).

|  | ICU-AW | Delirium |
| --- | --- | --- |
| **Predisposing Factors** | | |
| **Age** | DS^1–4^ | **SR** ^5–9^ |
| **Premorbid dementia** |  | **SR** ^10–13^ |
| Hypertension |  | DS ^14,15^ |
| Alcoholism |  | DS^6,10,14,16^ |
| **Prior Coma** |  | **SR** ^8,14,17,18^ |
| Nicotine Use |  | DS ^15,16,19^ |
| **Sex** | **SR**^20^ |  |
| **Admission APACHE II** | **SR** ^1–3,21^ | **SR** ^11,18,22–24^ |
| **Emergency Surgery and Trauma** |  | **SR** ^17,25^ |
| **Risk Factors** | | |
| Prolonged immobilization | DS ^4,20,26–27^ |  |
| **Blood Transfusion** |  | **SR** ^8^ |
| Improper use of Benzodiazepines and Opiates |  | DS ^7,13,18,23,24,28–31^ |
| **NMBAs** | **SR** ^1,3,32,33^ | DS ^29^ |
| **Aminoglycosides** | **SR** ^4,34–36^ |  |
| **Sepsis** | **SR** ^1,3,33,36,37^ | DS ^6,8,38,39^ |
| **MOF** | **SR** ^20,36,40^ | DS ^6,8,38^ |
| **Vasoactive Agents** | **SR** ^4,41^ |  |
| Corticosteroids | DS ^1,20,42^ |  |
| RRT | DS ^1,3,36,37^ |  |
| Gender | DS ^20^ |  |
| **Mechanical Ventilation** | **SR** ^20^ | DS ^43,44^ |
| **Electrolytes and metabolic disturbance** | **SR** ^5,18,45^ | DS ^5^ |
| **Hyperosmolarity** | **SR** ^36^ |  |
| **Hyperglycemia** | **SR** ^20,35,46,47^ |  |
| **High lactate** | **SR** ^34^ |  |
| Sleep deprivation |  | DS ^48^ |
| **Parenteral nutrition** | **SR** ^36^ |  |
| **Short Term Outcomes** | | |
| **Prolonged mechanical Ventilation** | DS ^20,32,49^ | DS ^16,39,50,51^ |
| **Prolonged ICU-LOS and H-LOS** | DS ^26^ | DS ^52,53^ |
| **ICU and H Mortality** | DS ^54,55^ | DS ^39,52,53^ |
|  |  |  |
| **Long Term Outcomes** | | |
| **Mortality** | DS ^32,54,55^ | DS ^56–58^ |
| **Long-term physical function impairment** | DS ^26,42,59–61^ | DS ^62^ |
| **Long-term cognitive impairment** | DS ^26,42,51,61^ | DS ^51,63–66^ |

Systematic Review (SR) indicates that the predisposing factors and trigger factors are in agreement with the Prevention and Management of Pain, Agitation/Sedation, Delirium, Immobility, and Sleep Disruption in Adult Patients in the ICU (PADIS) guidelines^67^ and a recent systematic review for ICU-AW^68^. Descriptive Study (DS) indicates that the predisposing factors and trigger factors are in agreement with single observation cohort or case-control studies or narrative reviews.

H, Hospital; LOS, Length of stay; MOF, Multi organ failure; NMBAs, Neuromuscular blocking agents; RRT, Renal replacement therapy.

**References associated with supplementary Table 1.**

1. Hermans G, Casaer MP, Clerckx B, Güiza F, Vanhullebusch T, Derde S, Meersseman P, Derese I, Mesotten D, Wouters PJ, van Cromphaut S, Debaveye Y, Gosselink R, Gunst J, Wilmer A, van den Berghe G, Vanhorebeek I. **Effect of tolerating macronutrient deficit on the development of intensive-care unit acquired weakness: A subanalysis of the EPaNIC trial.** *The Lancet Respiratory Medicine* 2013;**1**:621–9. doi:10.1016/S2213-2600(13)70183-8.
2. Patel BK, Pohlman AS, Hall JB, Kress JP. **Impact of early mobilization on glycemic control and ICU-acquired weakness in critically ill patients who are mechanically ventilated.** *Chest* 2014;**146**:583–9. doi:10.1378/chest.13-2046.
3. Hermans G, Wilmer A, Meersseman W, Milants I, Wouters PJ, Bobbaers H, Bruyninckx F, van den Berghe G. **Impact of Intensive Insulin Therapy on Neuromuscular Complications and Ventilator Dependency in the Medical Intensive Care Unit.** *Am J Respir Crit Care Med* 2007;**175**:480–9. doi:10.1164/rccm.200605-665OC.
4. van den Berghe G, Schoonheydt K, Becx P, Bruyninckx F, Wouters PJ. **Insulin therapy protects the central and peripheral nervous system of intensive care patients.** *Neurology* 2005;**64**:1348–53. doi:10.1212/01.WNL.0000158442.08857.FC.
5. Aldemir M, Ozen S, Kara IH, Sir A, Baç B. **Predisposing factors for delirium in the surgical intensive care unit.** *Crit Care* 2001;**5**:265–70.
6. Heymann A, Sander M, Krahne D, Deja M, Weber-Carstens S, MacGuill M, Kastrup M, Wernecke KD, Nachtigall I, Spies CD. **Hyperactive delirium and blood glucose control in critically ill patients.** *J Int Med Res* 2007;**35**:666–77. doi:10.1177/147323000703500511.
7. Colombo R, Corona A, Praga F, Minari C, Giannotti C, Castelli A, Raimondi F. **A reorientation strategy for reducing delirium in the critically ill. Results of an interventional study.** *Minerva Anestesiol* 2012;**78**:1026–33.
8. Angles EM, Robinson TN, Biffl WL, Johnson J, Moss M, Tran ZV, Moore EE. **Risk factors for delirium after major trauma.** *Am J Surg* 2008;**196**:864-9; discussion 869-70. doi:10.1016/j.amjsurg.2008.07.037.
9. Shehabi Y, Chan L, Kadiman S, Alias A, Ismail WN, Tan MATI, Khoo TM, Ali SB, Saman MA, Shaltut A, Tan CC, Yong CY, Bailey M. **Sedation depth and long-term mortality in mechanically ventilated critically ill adults: A prospective longitudinal multicentre cohort study.** *Intensive Care Med* 2013;**39**:910–8. doi:10.1007/s00134-013-2830-2.
10. van Rompaey B, Elseviers MM, Schuurmans MJ, Shortridge-Baggett LM, Truijen S, Bossaert L. **Risk factors for delirium in intensive care patients: A prospective cohort study.** *Crit Care* 2009;**13**:R77. doi:10.1186/cc7892.
11. Inouye SK, Zhang Y, Jones RN, Kiely DK, Yang F, Marcantonio ER. **Risk Factors for Delirium at Discharge.** *Arch Intern Med* 2007;**167**:1406. doi:10.1001/archinte.167.13.1406.
12. McNicoll L, Pisani MA, Zhang Y, Ely EW, Siegel MD, Inouye SK. **Delirium in the Intensive Care Unit: Occurrence and Clinical Course in Older Patients.** *J Am Geriatr Soc* 2003;**51**:591–8. doi:10.1034/j.1600-0579.2003.00201.x.
13. Agarwal V, O'Neill PJ, Cotton BA, Pun BT, Haney S, Thompson J, Kassebaum N, Shintani A, Guy J, Ely EW, Pandharipande P. **Prevalence and risk factors for development of delirium in burn intensive care unit patients.** *J Burn Care Res* 2010;**31**:706–15. doi:10.1097/BCR.0b013e3181eebee9.
14. Ouimet S, Kavanagh BP, Gottfried SB, Skrobik Y. **Incidence, risk factors and consequences of ICU delirium.** *Intensive Care Med* 2007;**33**:66–73. doi:10.1007/s00134-006-0399-8.
15. Dubois M-J, Bergeron N, Dumont M, Dial S, Skrobik Y. **Delirium in an intensive care unit: A study of risk factors.** *Intensive Care Med* 2001;**27**:1297–304. doi:10.1007/s001340101017.
16. Mehta S, Cook D, Devlin JW, Skrobik Y, Meade M, Fergusson D, Herridge M, Steinberg M, Granton J, Ferguson N, Tanios M, Dodek P, Fowler R, Burns K, Jacka M, Olafson K, Mallick R, Reynolds S, Keenan S, Burry L. **Prevalence, risk factors, and outcomes of delirium in mechanically ventilated adults.** *Crit Care Med* 2015;**43**:557–66. doi:10.1097/CCM.0000000000000727.
17. Serafim RB, Dutra MF, Saddy F, Tura B, Castro JEC de, Villarinho LC, da Gloria Santos M, Bozza FA, Rocco JR. **Delirium in postoperative nonventilated intensive care patients: Risk factors and outcomes.** *Ann Intensive Care* 2012;**2**:51. doi:10.1186/2110-5820-2-51.
18. Boogaard Mvd, Pickkers P, Slooter AJC, Kuiper MA, Spronk PE, Voort PHJvd, Hoeven JGvd, Donders R, Achterberg Tv, Schoonhoven L. **Development and validation of PRE-DELIRIC (PREdiction of DELIRium in ICu patients) delirium prediction model for intensive care patients: Observational multicentre study.** *BMJ* 2012;**344**:e420-e420. doi:10.1136/bmj.e420.
19. van Rompaey B, Elseviers MM, van Drom W, Fromont V, Jorens PG. **The effect of earplugs during the night on the onset of delirium and sleep perception: A randomized controlled trial in intensive care patients.** *Crit Care* 2012;**16**:R73. doi:10.1186/cc11330.
20. Jonghe B de, Sharshar T, Lefaucheur J-P, Authier F-J, Durand-Zaleski I, Boussarsar M, Cerf C, Renaud E, Mesrati F, Carlet J, Raphaël J-C, Outin H, Bastuji-Garin S. **Paresis acquired in the intensive care unit: A prospective multicenter study.** *JAMA: The Journal of the American Medical Association* 2002;**288**:2859–67.
21. Weber-Carstens S, Deja M, Koch S, Spranger J, Bubser F, Wernecke KD, Spies CD, Spuler S, Keh D. **Risk factors in critical illness myopathy during the early course of critical illness: A prospective observational study.** *Crit Care* 2010;**14**:R119. doi:10.1186/cc9074.
22. Yoshitaka S, Egi M, Morimatsu H, Kanazawa T, Toda Y, Morita K. **Perioperative plasma melatonin concentration in postoperative critically ill patients: Its association with delirium.** *J Crit Care* 2013;**28**:236–42. doi:10.1016/j.jcrc.2012.11.004.
23. Pandharipande P, Shintani A, Peterson J, Pun BT, Wilkinson GR, Dittus RS, Bernard GR, Ely EW. **Lorazepam Is an Independent Risk Factor for Transitioning to Delirium in Intensive Care Unit Patients.** *Anesthesiology* 2006;**104**:21–6. doi:10.1097/00000542-200601000-00005.
24. Pandharipande PP, Morandi A, Adams JR, Girard TD, Thompson JL, Shintani AK, Ely EW. **Plasma tryptophan and tyrosine levels are independent risk factors for delirium in critically ill patients.** *Intensive Care Med* 2009;**35**:1886–92. doi:10.1007/s00134-009-1573-6.
25. Veiga D, Luis C, Parente D, Fernandes V, Botelho M, Santos P, Abelha F. **Postoperative Delirium in Intensive Care Patients: Risk Factors and Outcome.** *Brazilian Journal of Anesthesiology* 2012;**62**:469–83. doi:10.1016/S0034-7094(12)70146-0.
26. Fan E, Dowdy DW, Colantuoni E, Mendez-Tellez PA, Sevransky JE, Shanholtz C, Himmelfarb CRD, Desai SV, Ciesla N, Herridge MS, Pronovost PJ, Needham DM. **Physical complications in acute lung injury survivors: A two-year longitudinal prospective study.** *Crit Care Med* 2014;**42**:849–59. doi:10.1097/CCM.0000000000000040.
27. Witt NJ, Zochodne DW, Bolton CF, Grand'Maison F, Wells G, Young GB, Sibbald WJ. **Peripheral Nerve Function in Sepsis and Multiple Organ Failure.** *Chest* 1991;**99**:176–84. doi:10.1378/chest.99.1.176.
28. Dunn WF, Adams SC, Adams RW. **Iatrogenic delirium and coma: A "near miss".** *Chest* 2008;**133**:1217–20. doi:10.1378/chest.08-0471.
29. Pandharipande P, Cotton BA, Shintani A, Thompson J, Pun BT, Morris JA, Dittus R, Ely EW. **Prevalence and Risk Factors for Development of Delirium in Surgical and Trauma Intensive Care Unit Patients.** *J Trauma* 2008;**65**:34–41. doi:10.1097/TA.0b013e31814b2c4d.
30. Seymour CW, Pandharipande PP, Koestner T, Hudson LD, Thompson JL, Shintani AK, Ely EW, Girard TD. **Diurnal sedative changes during intensive care: Impact on liberation from mechanical ventilation and delirium.** *Crit Care Med* 2012;**40**:2788–96. doi:10.1097/CCM.0b013e31825b8ade.
31. Andreasen A, Pedersen-Skovsgaard T, Mortensen O, van Hall G, Moseley P, Pedersen B. **The effect of glutamine infusion on the inflammatory response and HSP70 during human experimental endotoxaemia.** *Crit Care* 2009;**13**:R7. doi:10.1186/cc7696.
32. Hermans G, van Mechelen H, Clerckx B, Vanhullebusch T, Mesotten D, Wilmer A, Casaer MP, Meersseman P, Debaveye Y, van Cromphaut S, Wouters PJ, Gosselink R, van den Berghe G. **Acute Outcomes and 1-Year Mortality of Intensive Care Unit–acquired Weakness. A Cohort Study and Propensity-matched Analysis.** *Am J Respir Crit Care Med* 2014;**190**:410–20. doi:10.1164/rccm.201312-2257OC.
33. Brunello A-G, Haenggi M, Wigger O, Porta F, Takala J, Jakob SM. **Usefulness of a clinical diagnosis of ICU-acquired paresis to predict outcome in patients with SIRS and acute respiratory failure.** *Intensive Care Med* 2010;**36**:66–74. doi:10.1007/s00134-009-1645-7.
34. Wieske L, Witteveen E, Verhamme C, Dettling-Ihnenfeldt DS, van der Schaaf M, Schultz MJ, van Schaik IN, Horn J, Salluh JIF. **Early Prediction of Intensive Care Unit–Acquired Weakness Using Easily Available Parameters: A Prospective Observational Study.** *PLoS ONE* 2014;**9**:e111259. doi:10.1371/journal.pone.0111259.
35. Nanas S, Kritikos K, Angelopoulos E, Siafaka A, Tsikriki S, Poriazi M, Kanaloupiti D, Kontogeorgi M, Pratikaki M, Zervakis D, Routsi C, Roussos C. **Predisposing factors for critical illness polyneuromyopathy in a multidisciplinary intensive care unit.** *Acta Neurol Scand* 2008;**118**:175–81. doi:10.1111/j.1600-0404.2008.00996.x.
36. Garnacho-Montero J, Madrazo-Osuna J, García-Garmendia J, Ortiz-Leyba C, Jiménez-Jiménez F, Barrero-Almodóvar A, Garnacho-Montero M, Moyano-Del-Estad M. **Critical illness polyneuropathy: Risk factors and clinical consequences. A cohort study in septic patients.** *Intensive Care Med* 2001;**27**:1288–96. doi:10.1007/s001340101009.
37. van den Berghe G, Wouters P, Weekers F, Verwaest C, Bruyninckx F, Schetz M, Vlasselaers D, Ferdinande P, Lauwers P, Bouillon R. **Intensive insulin therapy in critically ill patients.** *N Engl J Med* 2001;**345**:1359–67. doi:10.1056/NEJMoa011300.
38. Pandharipande PP, Pun BT, Herr DL, Maze M, Girard TD, Miller RR, Shintani AK, Thompson JL, Jackson JC, Deppen SA, Stiles RA, Dittus RS, Bernard GR, Ely EW. **Effect of sedation with dexmedetomidine vs lorazepam on acute brain dysfunction in mechanically ventilated patients: The MENDS randomized controlled trial.** *JAMA* 2007;**298**:2644–53. doi:10.1001/jama.298.22.2644.
39. Lin S-M, Huang C-D, Liu C-Y, Lin H-C, Wang C-H, Huang P-Y, Fang Y-F, Shieh M-H, Kuo H-P. **Risk factors for the development of early-onset delirium and the subsequent clinical outcome in mechanically ventilated patients.** *J Crit Care* 2008;**23**:372–9. doi:10.1016/j.jcrc.2006.09.001.
40. Bednark J, Vondracek P, Dusek L, Moravcova E, Cundrle I. **Risk factors for critical illness polyneuromyopathy.** *J Neurol* 2005;**252**:343–51. doi:10.1007/s00415-005-0654-x.
41. Wolfe KS, Patel BK, MacKenzie EL, Giovanni SP, Pohlman AS, Churpek MM, Hall JB, Kress JP. **Impact of Vasoactive Medications on ICU-Acquired Weakness in Mechanically Ventilated Patients.** *Chest* 2018;**154**:781–7. doi:10.1016/j.chest.2018.07.016.
42. Herridge MS, Cheung AM, Tansey CM, Matte-Martyn A, Diaz-Granados N, Al-Saidi F, Cooper AB, Guest CB, Mazer CD, Mehta S, Stewart TE, Barr A, Cook D, Slutsky AS. **One-Year Outcomes in Survivors of the Acute Respiratory Distress Syndrome.** *N Engl J Med* 2003;**348**:683–93. doi:10.1056/NEJMoa022450.
43. Tilouche N, Hassen MF, Ali HBS, Jaoued O, Gharbi R, El Atrous SS. **Delirium in the Intensive Care Unit: Incidence, risk factors, and impact on outcome.** *Indian Journal of Critical Care Medicine* 2018;**22**:144–9. doi:10.4103/ijccm.IJCCM_244_17.
44. Kim H, Chung S, Joo YH, Lee JS. **The major risk factors for delirium in a clinical setting.** *NDT* 2016;**12**:1787–93. doi:10.2147/NDT.S112017.
45. Nguyen The L, Nguyen Huu C. **Critical illness polyneuropathy and myopathy in a rural area in Vietnam.** *J Neurol Sci* 2015;**357**:276–81. doi:10.1016/j.jns.2015.08.005.
46. Bercker S, Weber-Carstens S, Deja M, Grimm C, Wolf S, Behse F, Busch T, Falke KJ, Kaisers U. **Critical illness polyneuropathy and myopathy in patients with acute respiratory distress syndrome*.** *Crit Care Med* 2005;**33**:711–5. doi:10.1097/01.CCM.0000157969.46388.A2.
47. Hermans G, Schrooten M, van Damme P, Berends N, Bouckaert B, Vooght W de, Robberecht W, van den Berghe G. **Benefits of intensive insulin therapy on neuromuscular complications in routine daily critical care practice: A retrospective study.** *Crit Care* 2009;**13**:R5. doi:10.1186/cc7694.
48. Fadayomi AB, Ibala R, Bilotta F, Westover MB, Akeju O. **A Systematic Review and Meta-Analysis Examining the Impact of Sleep Disturbance on Postoperative Delirium.** *Crit Care Med* 2018;**46**:e1204-e1212. doi:10.1097/CCM.0000000000003400.
49. Jonghe B de, Bastuji-Garin S, Durand M-C, Malissin I, Rodrigues P, Cerf C, Outin H, Sharshar T. **Respiratory weakness is associated with limb weakness and delayed weaning in critical illness*.** *Crit Care Med* 2007;**35**:2007–15. doi:10.1097/01.ccm.0000281450.01881.d8.
50. van den Boogaard M, Schoonhoven L, van der Hoeven JG, van Achterberg T, Pickkers P. **Incidence and short-term consequences of delirium in critically ill patients: A prospective observational cohort study.** *Int J Nurs Stud* 2012;**49**:775–83. doi:10.1016/j.ijnurstu.2011.11.016.
51. van den Boogaard M, Schoonhoven L, Evers AWM, van der Hoeven JG, van Achterberg T, Pickkers P. **Delirium in critically ill patients: Impact on long-term health-related quality of life and cognitive functioning.** *Crit Care Med* 2012;**40**:112–8. doi:10.1097/CCM.0b013e31822e9fc9.
52. Ely EW, Shintani A, Truman B, Speroff T, Gordon SM, Harrell FE, Inouye SK, Bernard GR, Dittus RS. **Delirium as a predictor of mortality in mechanically ventilated patients in the intensive care unit.** *JAMA* 2004;**291**:1753–62. doi:10.1001/jama.291.14.1753.
53. Ely E, Gautam S, Margolin R, Francis J, May L, Speroff T, Truman B, Dittus R, Bernard G, Inouye S. **The impact of delirium in the intensive care unit on hospital length of stay.** *Intensive Care Med* 2001;**27**:1892–900. doi:10.1007/s00134-001-1132-2.
54. Ali NA, O'Brien JM, Hoffmann SP, Phillips G, Garland A, Finley JCW, Almoosa K, Hejal R, Wolf KM, Lemeshow S, Connors AF, Marsh CB. **Acquired weakness, handgrip strength, and mortality in critically ill patients.** *Am J Respir Crit Care Med* 2008;**178**:261–8. doi:10.1164/rccm.200712-1829OC.
55. Sharshar T, Bastuji-Garin S, Stevens RD, Durand M-C, Malissin I, Rodriguez P, Cerf C, Outin H, Jonghe B de. **Presence and severity of intensive care unit-acquired paresis at time of awakening are associated with increased intensive care unit and hospital mortality.** *Crit Care Med* 2009;**37**:3047–53. doi:10.1097/CCM.0b013e3181b027e9.
56. McCusker J, Cole M, Abrahamowicz M, Primeau F, Belzile E. **Delirium Predicts 12-Month Mortality.** *Arch Intern Med* 2002;**162**:457. doi:10.1001/archinte.162.4.457.
57. Pisani MA, Kong SYJ, Kasl SV, Murphy TE, Araujo KLB, van Ness PH. **Days of delirium are associated with 1-year mortality in an older intensive care unit population.** *Am J Respir Crit Care Med* 2009;**180**:1092–7. doi:10.1164/rccm.200904-0537OC.
58. Thomason JWW, Shintani A, Peterson JF, Pun BT, Jackson JC, Ely EW. **Intensive care unit delirium is an independent predictor of longer hospital stay: A prospective analysis of 261 non-ventilated patients.** *Crit Care* 2005;**9**:R375-81. doi:10.1186/cc3729.
59. Wieske L, Dettling-Ihnenfeldt DS, Verhamme C, Nollet F, van Schaik IN, Schultz MJ, Horn J, van der Schaaf M. **Impact of ICU-acquired weakness on post-ICU physical functioning: A follow-up study.** *Crit Care* 2015;**19**:502. doi:10.1186/s13054-015-0937-2.
60. Latronico N, Shehu I, Seghelini E. **Neuromuscular sequelae of critical illness.** *Curr Opin Crit Care* 2005;**11**:381–90. doi:10.1097/01.ccx.0000168530.30702.3e.
61. Herridge MS, Tansey CM, Matté A, Tomlinson G, Diaz-Granados N, Cooper A, Guest CB, Mazer CD, Mehta S, Stewart TE, Kudlow P, Cook D, Slutsky AS, Cheung AM. **Functional disability 5 years after acute respiratory distress syndrome.** *N Engl J Med* 2011;**364**:1293–304. doi:10.1056/NEJMoa1011802.
62. O'Keeffe S, Lavan J. **The Prognostic Significance of Delirium in Older Hospital Patients.** *J Am Geriatr Soc* 1997;**45**:174–8. doi:10.1111/j.1532-5415.1997.tb04503.x.
63. Girard TD, Jackson JC, Pandharipande PP, Pun BT, Thompson JL, Shintani AK, Gordon SM, Canonico AE, Dittus RS, Bernard GR, Ely EW. **Delirium as a predictor of long-term cognitive impairment in survivors of critical illness.** *Crit Care Med* 2010;**38**:1513–20. doi:10.1097/CCM.0b013e3181e47be1.
64. Wolters AE, van Dijk D, Pasma W, Cremer OL, Looije MF, Lange DW de, Veldhuijzen DS, Slooter AJC. **Long-term outcome of delirium during intensive care unit stay in survivors of critical illness: A prospective cohort study.** *Crit Care* 2014;**18**:R125. doi:10.1186/cc13929.
65. Girard TD, Thompson JL, Pandharipande PP, Brummel NE, Jackson JC, Patel MB, Hughes CG, Chandrasekhar R, Pun BT, Boehm LM, Elstad MR, Goodman RB, Bernard GR, Dittus RS, Ely EW. **Clinical phenotypes of delirium during critical illness and severity of subsequent long-term cognitive impairment: A prospective cohort study.** *The Lancet Respiratory Medicine* 2018;**6**:213–22. doi:10.1016/S2213-2600(18)30062-6.
66. Witlox J, Eurelings LSM, Jonghe JFM de, Kalisvaart KJ, Eikelenboom P, van Gool WA. **Delirium in elderly patients and the risk of postdischarge mortality, institutionalization, and dementia: A meta-analysis.** *JAMA* 2010;**304**:443–51. doi:10.1001/jama.2010.1013.
67. Devlin JW, Skrobik Y, Gélinas C, Needham DM, Slooter AJC, Pandharipande PP, Watson PL, Weinhouse GL, Nunnally ME, Rochwerg B, Balas MC, van den Boogaard M, Bosma KJ, Brummel NE, Chanques G, Denehy L, Drouot X, Fraser GL, Harris JE, Joffe AM, Kho ME, Kress JP, Lanphere JA, McKinley S, Neufeld KJ, Pisani MA, Payen J-F, Pun BT, Puntillo KA, Riker RR, Robinson BRH, Shehabi Y, Szumita PM, Winkelman C, Centofanti JE, Price C, Nikayin S, Misak CJ, Flood PD, Kiedrowski K, Alhazzani W. **Clinical Practice Guidelines for the Prevention and Management of Pain, Agitation/Sedation, Delirium, Immobility, and Sleep Disruption in Adult Patients in the ICU.** *Crit Care Med* 2018;**46**:e825-e873. doi:10.1097/CCM.0000000000003299.
68. Yang T, Li Z, Jiang L, Wang Y, Xi X. **Risk factors for intensive care unit-acquired weakness: A systematic review and meta-analysis.** *Acta Neurol Scand* 2018;**138**:104–14. doi:10.1111/ane.12964.
